# Exploring Pregnant Women’s Perceptions and Experiences of Adiposity Measurements in Routine Antenatal Care: A Qualitative Study

**DOI:** 10.3390/healthcare13202558

**Published:** 2025-10-10

**Authors:** Susan C. Lennie, Luke Vale, M. Dawn Teare, Raya Vinogradov, Nicola Heslehurst

**Affiliations:** 1 Population Health Sciences Institute, Newcastle University, Newcastle upon Tyne NE2 4HH, UK; luke.vale@newcastle.ac.uk (L.V.); dawn.teare@newcastle.ac.uk (M.D.T.); raya.vinogradov@newcastle.ac.uk (R.V.); nicola.heslehurst@newcastle.ac.uk (N.H.); 2 School of Biomedical, Nutritional and Sport Sciences, Faculty of Medical Sciences, Newcastle University, Dame Margaret Barbour Building, Newcastle upon Tyne NE2 4DR, UK; 3 Department of Health Services Research and Policy, London School of Hygiene and Tropical Medicine, London WC1E 7HT, UK; 4 Research Directorate, Newcastle upon Tyne Hospitals NHS Foundation Trust, Newcastle upon Tyne NE1 4LP, UK

**Keywords:** antenatal care, anthropometry, body composition measurement, maternal adiposity, theoretical framework of acceptability (TFA)

## Abstract

**Background/objectives:** Maternal adiposity is a known risk factor for adverse pregnancy outcomes, yet routine antenatal care primarily relies on body mass index (BMI), which has limitations. This study aimed to explore the acceptability of incorporating a broader range of adiposity measurements into early pregnancy antenatal care, assessing pregnant women’s perceptions to inform implementation strategies. **Methods:** A qualitative study using semi-structured interviews was conducted with 14 pregnant women purposively sampled to capture variation in BMI, age, and parity. Interviews occurred approximately 4–5 months post-measurement experience. The Theoretical Framework of Acceptability (TFA) guided thematic analysis of transcribed data, with independent coding to ensure rigour. **Results**: Participants generally viewed the current reliance on BMI as outdated and expressed neutral to positive attitudes toward the use of more detailed adiposity measurements. Most reported little emotional discomfort with the process. However, some reflected likelihood of more body self-consciousness had it been their first pregnancy. Time involved in measurements was not seen as burdensome, however waiting between procedures was a minor inconvenience. Self-assessing body shape was described as difficult. Women emphasised the importance of choice, autonomy, and informed consent, especially in relation to partner involvement, the gender of the anthropometrist, and the nature of the procedures. Clear, advance communication and supportive explanations during appointments were seen as essential to ensuring a positive experience. **Conclusions:** Expanding adiposity assessments in early pregnancy is acceptable to women if implemented ethically, prioritising consent, privacy, emotional safety, and effective communication. Integration into routine care requires staff training and pre-appointment guidance.

## 1. Introduction

Body mass and stature are fundamental anthropometric measures serving as key indicators for establishing baseline health profiles, assessing nutritional status, informing critical clinical decisions and optimising medication dosages [1,2,3].

Existing maternity clinical guidelines recommend measuring body mass index (BMI) in early pregnancy (as a proxy for pre-pregnancy BMI) [4] to classify women at risk for obesity-related adverse pregnancy outcomes (BMI ≥ 30.0 kg/m^2^) [5]. In the UK, women with early pregnancy BMI ≥ 30.0 kg/m^2^ receive extra screening/monitoring, referral for specialist care, and delivery in an obstetric unit with additional equipment and expertise [6,7]. Public Health England [8] reported that in 2017, 21.6% of >540,000 pregnant women had BMI ≥ 30.0 kg/m^2^.

Whilst BMI can, on a population basis, monitor patterns in population-level risk, it has poor sensitivity for detection of excess body fat and health risk on an individual level [9]. Alternative body fat distribution measures provide more accurate estimates of individual risk [10]. In pregnancy, using BMI to determine individual risk can result in needless clinical intervention for some women, and mis-classify risk for women with high adiposity but a BMI < 30 kg/m^2^ [11]. In a scoping review of the experiences of antenatal maternity care of women with obesity, Saw et al. [12] identified numerous studies reporting a perception of being subjected to over-medicalisation and excessive monitoring, leading to anxiety and depersonalised care.

Alternative anthropometric measures that may more accurately assess individual risk for adverse pregnancy outcomes are being explored in the SHAPES study [13]. This is a prospective cohort study at Newcastle upon Tyne Hospitals NHS Trust that recruited 1450 pregnant women at their first trimester ultrasound scan (11^+2^–14^+1^ weeks’) with the aim to evaluate whether adiposity measures during early pregnancy, either alone or in combination with other factors, better predict pregnancy complications than BMI. The study data includes measurement of waist, hip, mid-upper arm, and neck circumferences, skinfold thicknesses at several sites (subscapular, triceps, biceps, and iliac crest, and supraspinale), abdominal and pre-peritoneal subcutaneous and visceral adipose tissue measurements via ultrasound, as well as self-identification of body shape utilising subjective visual tools [14,15]. By exploring a comprehensive range of adiposity measures, the SHAPES study seeks to address the known limitations of BMI as a predictor of adverse maternal and infant outcomes and provide evidence to support improved clinical risk stratification.

However, the adoption of an alternative adiposity measurement into routine care requires more than just data on predictive performance. It is crucial to consider how these potentially unfamiliar measurements are perceived by pregnant women, as well as the potential impact on their maternity care experience.

Some expectant mothers feel apprehension, concern, and dissatisfaction related to weight gain and physical changes during pregnancy [16,17]. However, other women experience increased body self-esteem and a positive attitude to these changes [18,19,20], with factors such as pre-pregnancy exercise behaviour being influential [21]. Furthermore, there is evidence that body image dissatisfaction in early pregnancy significantly predicts ongoing dissatisfaction with body image a year later [22]. Weight stigma and body dissatisfaction may therefore be important considerations in shaping how routine adiposity-related assessments are implemented within antenatal care policy and practice.

Previous qualitative research [23,24] exploring the experiences of midwives delivering maternal obesity care, suggests that measurement practices may be compromised because of anxieties on the part of both the midwife and the pregnant woman. In Heslehurst et al. [24], one midwife reflected that “*Every time she comes in, she is completely totally embarrassed that I have to look and feel her tummy… you can see her almost like, fear factor that I’m gonna mention something, and I find then that situation much harder because I don’t wanna hurt her*”. Research [12] suggests that weight status is not addressed, and Atkinson et al. [25] refer to this as an ‘unconscious collusion’ between healthcare providers and women to avoid difficult or sensitive topics. An important consideration in potentially extending the range of anthropometric measurements in antenatal care, therefore, is the perception and experience of the woman, as well as the confidence of the professional, and how measurement results are communicated.

The aim of this study was to qualitatively explore women’s perception, experiences and retrospective acceptability of adiposity measurements during routine dating scan appointments. To provide a clear focus for the study, the following research questions were developed to align with this aim: (1) How do women perceive adiposity measurements during routine dating scan appointments? (2) What are women’s experiences of undergoing these measurements? (3) How acceptable do women find these measurements in retrospect?

## 2. Materials and Methods

This study used an interpretive constructivist approach, where there is an understanding that individuals’ perspectives of situations are constructed through their interactions, and aims to understand experiences from individuals’ perspective [26]. This approach facilitated the exploration of having additional anthropometric measurements taken during routine National Health Service (NHS) antenatal ultrasound scan appointments based on the participants experience of this interaction.

### 2.1. Recruitment and Sampling

Participants were recruited from the SHAPES cohort study (April 2022–April 2024). On enrolment, SHAPES participants were asked if they were willing to be contacted for an interview study. Interview recruitment was conducted between June-September 2024, targeting those recruited to SHAPES between January–April 2024 (n = 220) to minimise time from measurement to interview. A purposive, maximum-variation sampling strategy was employed to aim for diversity of demographics (BMI, age, parity, and ethnicity). One hundred eligible women were screened and those who had miscarried, shown warning signs of miscarriage, or experienced stillbirth or neonatal death were excluded (n = 5). Ninety-five were contacted by email with a participant information sheet. Twenty-seven responded. Data saturation was reached when no new codes were identified. Upon interview completion, the participant received a GBP 15 shopping voucher.

### 2.2. Data Collection

Semi-structured interviews were conducted via Microsoft Teams. This format was selected to facilitate in-depth exploration of participants’ personal perceptions and emotional responses to the additional measurements whilst also enabling the interviewer to follow up on responses and probe emerging themes. This method aligns with the interpretive constructivist approach by allowing participants to express nuanced views in their own words and provided a private space to discuss potentially sensitive topics such as body image, measurement discomfort, and prior healthcare experiences. Individual interviews were preferred over focus groups to reduce social desirability bias and avoid the influence of group dynamics on participants’ willingness to share openly [27].

Interviews were conducted by the same interviewer (SL) using a flexible topic guide, with consideration of principles of good practice in anthropometry from the International Society for the Advancement of Kinanthropometry (ISAK) [28]. The guide covered topics such as previous body measurement experiences, participant expectations, and the acceptability of the additional measurements, including the sensory aspects of measurement, environment, and the etiquette involved (Document S1: Topic Guide). Interviews lasted approximately 30 min. All interviews were audio- or video-recorded and transcribed verbatim using Microsoft Teams transcription software. Transcripts were de-identified, checked for accuracy by the researcher and videos were deleted following transcription.

### 2.3. SHAPES Appointment Structure and Setting

To aid interpretation of participants’ experiences, the following provides an overview of the structure and setting of the SHAPES appointment in which the adiposity measurements occurred. Anthropometric measurements were conducted during participants’ routine dating scan appointments, which also included blood tests. The ultrasound scan typically took place first, followed by the other procedures in no fixed order. The anthropometry measurements we completed by a researcher trained in anthropometry, and took place in a repurposed office, measuring approximately 2.5 m by 3 m, located along a short corridor adjacent to the antenatal clinic. Participants were required to move between rooms for the different procedures, sometimes waiting briefly in the waiting area.

### 2.4. Data Analysis

Audio-recordings were re-played and cross-checked with the transcripts to correct any transcription errors and support re-familiarisation with the data (SL). Interview transcripts were de-identified and imported into NVivo (version 1.7.1; QSR International) to support data management, and coding. Transcripts were coded line by line by one researcher (SL), with codes generated inductively from the data. To enhance the consistency and reliability of the coding process, two additional researchers (RV and NH) independently coded a single transcript for comparison. Following initial coding, a framework-informed thematic analysis was undertaken by mapping codes on to the seven constructs of the Theoretical Framework of Acceptability (TFA) [29] (Table 1). Subthemes were developed for each TFA construct to capture patterned meanings across the dataset. Codes that did not align with the TFA were retained and themed separately, to ensure important data outside the framework were not excluded. Participant characteristics were drawn from the SHAPES cohort study dataset following qualitative interviews with Index of Multiple Deprivation (IMD) decile identified using the participants postcode via the English indices of deprivation 2019 dataset (https://imd-by-postcode.opendatacommunities.org/imd/2019 accessed on 2 May 2025).

The analysis is supported by representative verbatim quotes from research participants to illustrate findings. Quotes are clearly indicated using inverted commas and italics with numbers in brackets after quotes indicate the corresponding participant ID. A reflexive diary was maintained to reflect on any potential bias, due to the researcher’s background in anthropometry and dietetics (SL).

The North East-Newcastle & North Tyneside 1 Research Ethics Committee approved the study protocol (REC reference: 22/NE/0035). Participants provided audio- or video-recorded informed verbal consent to participate and recording of interviews. Recordings of interviews were deleted following completion of the transcripts.

The Standards for Reporting Qualitative Research (SRQR) guidelines [30] were followed in our reporting (Table S1: SRQR guidelines).

## 3. Results

### 3.1. Participant Characteristics

Fourteen women aged between 26 and 35 years (mean 32.4 years) participated (Table 2). Their mean weight was 72.0 kg (range 50.0–105.0 kg) and mean BMI was 26.7 kg/m^2^ (range 17.4–35.6 kg/m^2^). Based on BMI classifications, five women were categorized as having obesity, two were classified as having overweight, six within the recommended range, and one with an underweight BMI. Eleven participants identified as White ethnicity, with the remaining individuals identifying as Black (n = 1), Asian (n = 1), or Mixed ethnic group (n = 1). The study population was broadly similar to those participating in SHAPES except for narrower age and BMI ranges. Most participants (n = 9) had at least one previous pregnancy, with parity ranging from 0 to 2.

### 3.2. Overview of Participant Perceptions

Overall, participants expressed largely neutral-positive views towards the adiposity measurements. Many described the experience as unproblematic or unmemorable, with comments such as: “*Absolutely fine, no issues*” [P10], and “*I don’t really remember them, so it’s probably saying to me that I was absolutely fine with them*” [P11]. Others acknowledged that while they were not personally bothered, the experience might have been more difficult for others: “*I didn’t mind at all. Some people may feel uncomfortable*” [P08]. However, the in-depth and probing nature of the interviews enabled the mapping of qualitative findings onto all seven constructs of the TFA [29], as well as the identification of one additional inductive theme and nineteen associated subthemes (Table 3). While the thematic analysis provides detailed insights into each construct, these should be interpreted within the broader context of generally neutral or modestly positive attitudes.

### 3.3. Affective Attitude

This construct contained four sub-themes: a positive orientation towards enhanced care; sensory and emotional responses to measurement procedures; internal discomfort: body image and vulnerability; and contextual discomfort: haptics and proxemics.

#### 3.3.1. Positive Orientation Towards Enhanced Care

Participants expressed positivity to the inclusion of a greater range of adiposity measures in maternal monitoring: “*I think it’s something that’s quite positive because it’s taking into consideration you individually, rather than your BMI*” (P01). This favourable outlook was often grounded in a belief that interventions contributing to better care were inherently worthwhile: “*I think you just go through anything that’s needed to get the best care really*” (P14).

#### 3.3.2. Sensory and Emotional Responses to Measurement Procedures

Participants described minimal emotional discomfort during measurement, though some reported mild sensory responses and unfamiliarity with specific procedures. Most women had only previously experienced weight, height, and girth assessments in healthcare, clothing fittings, slimming groups, fitness environments, or through self-monitoring. As a result, certain measures felt unfamiliar. The application of skinfold calipers, in particular, elicited sensations described as “pinching” or “nipping,” and feelings such as being “tickly,” or “uncomfortable but not painful.” These physical experiences were often accompanied by emotional reactions, as participants reflected on how the sensations made them feel. One participant reported emotional unease tied to unfamiliarity of the neck girth measurement, stating that “*It was strange. Someone putting anything around your neck. But no, it was absolutely fine. There was no concern.* […] *I think it’s just ‘cause you’re not used to somebody putting anything around your neck. It wasn’t in any way uncomfortable.*” (P10).

#### 3.3.3. Internal Discomfort: Body Image and Vulnerability

A minority of participants expressed affective responses rooted in body image sensitivities: *“It was a little bit uncomfortable for that reason… being self-conscious of the fact that you’re a little bit chubbier than what you’d like to be”* (P05). Others noted that the intimate nature of certain measurements could provoke feelings of discomfort or sensitivity, particularly during a first pregnancy: *“Like, I think that was quite, quite like intimate measurement, wasn’t it? Like you really had to get all of your parts out available to be measured physically”* (P13) and *“Maybe had it been my first pregnancy, I would’ve felt more uncomfortable about it”* (P02).

Drawing on broader experiences of working with diverse populations, one participant reflected: “*I suppose it’s kind of element of touching or, you know, undressing that sort of thing that might be quite sensitive*” (P01). Indeed, another participant noted that certain measurements could be distressing for individuals with histories of trauma: “*So just people that have experienced different kinds of abuse, like especially like sexual abuse, maybe physical abuse, I think. Taking the waist and the hip and the measurement from was it the back, or the just somewhere where you needed to be near like the bra area? I just feel like that could be triggering for some people*” (P06).

In relation to selecting a representative body shape from the subjective body shape assessment tools, participants generally found the task challenging, particularly due to changing body perceptions during pregnancy. One participant noted that it “*was the only thing I found a bit difficult*” (P03), highlighting the affective dimension of engaging with body image-based self-assessment.

#### 3.3.4. Contextual Discomfort: Haptics and Proxemics

The contextual discomfort relates to participants’ emotional responses to physical touch (haptics) and the perceived intrusion or maintenance of personal space (proxemics) during the measurement process. Some participants expressed that the experience of being touched by a stranger, even during pregnancy, could feel intrusive: “*I do think it’s a bit funny when, you know, people are touching you for any sort of reason, even for, like, you know, healthcare reasons.*” (P06). However, these feelings were often alleviated by the respectful and professional conduct of staff, including clear explanations of procedures and explicit consent-seeking prior to physical contact. For many, physical contact was seen as an expected or routine aspect of pregnancy care. Several participants explicitly contextualised the measurements within the broader experience of pregnancy, describing them as comparatively mild or insignificant: “*with going through it before, and they were always like touching bumps and feeling around and poking and prodding.* […] *That’s not the worst of it, is it? That’s fine*” (P14).

The importance of staff being considerate of proxemic boundaries also helped reduce mental discomfort: *“The person taking the measurement was very considerate, wasn’t obstructive of my face or my view.* […] *I didn’t have someone completely in my personal space. I think they were very conscientious of that”* (P10).

### 3.4. Burden

Participants’ generally perceived low burden associated with the intervention, with reflections centred around the subthemes of physical effort, time, and privacy.

#### 3.4.1. Physical Effort

Most participants described the physical aspects of the measurements as straightforward, with minimal disruption. However, participants often discussed the need to remove or adjust clothing which most accepted with ease: “*All the measurements could be taken with your clothes on, but a jumper kind of thing, you know, as long as there’s accessibility to the skin. But there was nothing that was particularly intrusive or anything*.” (P07). Occasional uncertainty about how much to adjust was quickly resolved by staff providing clear, reassuring instructions. Pre-appointment information also minimised physical effort: “*I think some of the documentation prior had sort of said wear something loose for your top that you know can be changed. And I think just knowing that they were taking measurements like I think I wore just sort of leggings or something that was appropriate for them to measure over.*” (P10).

Another potential physical burden was the use of pen to mark the skin for the purpose of accurate measurement location. Most reported that the marks were small, easily removed, and did not cause lasting discomfort. However, one participant mentioned feeling hesitation at the thought of having marks drawn on their skin, indicating a cognitive burden: “*I think I just do worry about oh, everything, like, I do get quite anxious. So, I was initially thinking, like, I hope this is safe for the baby, which is ridiculous. But I, yeah, I spoke to my partner afterwards and I think he reassured me. Like, it’s fine, it’s just pen*” (P06).

#### 3.4.2. Time Commitment and Waiting

Participants described the process as brief: “*Time wise, it didn’t take long. I think it took maybe 10 min or so*” (P02). However, the waiting, in contrast to the brevity of the measurements themselves, was occasionally noted as burdensome; “*[I spent] longer sitting waiting to be seen than having the measurements done*” (P02). Anticipated waiting was also seen as potentially burdensome: “*Yeah, I did think… am I gonna be waiting long for the other person to be ready?*” (P06).

Suggestions to reduce transitions and waiting were made, though participants acknowledged challenges with implementing these: *“if that it was a standard of care, then maybe the measurements being done at the same time as the scan, maybe in the scan room. But I doubt that would be something that would do.”* (P09)

#### 3.4.3. Privacy

A potential environmental contribution to burden was the degree of privacy afforded during measurements; however, participants reported that the private space used, particularly with a lockable door, mitigated this. The need for privacy was highlighted particularly when clothing needed to be adjusted or removed: “*I think the most important thing is, is making sure it feels private so you know somewhere you can shut the door and you’re not. Obviously, if you’re having to strip off your top or anything*” (P03). Suggestions like internal partitions were made to enhance comfort: “*You know like when you walk in, you go and have your scan.* […] *there’s the blue pull out wall thing.* […] *Maybe just something like that, I suppose. Just so there’s that little* […] *bit of extra privacy. Either healthcare professionals or just the door opening,* […] *Just having that space you can go to or if you need to remove or adjust some clothing*” (P11), though most reported the space felt sufficiently private.

Overall, participants described the intervention as requiring minimal effort across physical, cognitive, and logistical domains, with potential burdens mitigated by environmental and interpersonal factors.

### 3.5. Ethicality

Ethicality discussions centred on the limitations of BMI as a universal measure, and the interrelated concepts of choice, autonomy and consent.

#### 3.5.1. BMI as Ethically Problematic

Several participants questioned the ethicality of the current reliance on BMI, viewing it as an outdated, overly simplistic, and potentially stigmatising measure: “*I get a lot of frustration when it comes to BMI personally because I lift weights.* […] *I would never ever be able to achieve [*my BMI target*] without having like a really, really low body fat percentage.* […] *my background is sport, so I think it’s been the big shift in women’s participation in physical activity,* […] *which BMI hasn’t kept up with in terms of muscle mass.”* (P11). Indeed, the relevance during pregnancy was questioned: “*You don’t necessarily want to be told you’re obese when you’re just pregnant*” (P04). Support for more nuanced adiposity measurements designed for women during pregnancy was seen as a positive ethical development: “*I’m sure someone once said to me it’s based on like a white male as well. I was like well, why? If it’s women who are carrying babies like why are we looking at that?* […] *Why not update the system and have other measures out there*” (P03).

#### 3.5.2. Choice, Autonomy and Consent

Choice, autonomy and consent in maternal care was reflected in three key areas: participation of a partner or chaperone, sex/gender of the care provider, and the option to decline specific measurements.

Ten women involved a partner/chaperone in the anthropometry appointment, with the majority (n = 9) indicating that this was automatic and inclusive to the pregnancy experience. However, one participant emphasised the importance of choice and flexibility: “*Yeah, I think the option is important. I chose him to be there. But you know, at the end of the day, I might have been slightly more comfortable if he wasn’t to be honest, ‘cause* […] *you do feel a bit embarrassed about your measurements and stuff. […] I’m glad the option was there. It should be an option. It should be something that you can refuse as well*” (P03). One participant, who assumed their partner was not allowed to attend since this was not explicitly offered reflected that “*I think I probably would have felt a bit more comfortable had he been there, but I wasn’t, I wasn’t bothered enough to say anything*” (P06).

The presence of a partner or chaperone was also raised in the context of the sex/gender of the care provider: “*had it been a male doing the measurements, I might have felt a little bit uncomfortable if I was on my own”* (P02). While most indicated they would be personally comfortable with male staff, there was widespread agreement that others may not feel similarly and individuals should be offered a choice: “*I wouldn’t necessarily refuse a man taking the measurements, but I know there would be a lot of people out there that probably would, so I suppose it’s about patient choices*” (P09).

The option to decline specific measurements was also suggested. Participants felt ethical acceptability depended on being able to question the purpose of procedures and decline aspects they found unnecessary or intrusive: “*If, like, the routine care has gone without these measurements for so long, then if it’s optional, is that really a big issue?*” (P06).

Many participants described how their autonomy was supported through the consistent use of consent-seeking behaviours. Rather than obtaining consent as a one-time event, staff were perceived to check for permission throughout the process. “*They explained what they were going to do. They checked consent with me* […] *and made sure that I understood the information*” (P10).

### 3.6. Intervention Coherence

Within the theme of intervention coherence, subthemes related to the purpose of specific measures, and participants expectations of follow-up.

#### 3.6.1. Measurement Purpose

Most participants demonstrated an understanding of the measurement procedures: “*I knew what she was doing and why she was doing it*” (P03); however, some expressed uncertainty about the purpose and coherence of elements of the intervention. One participant expressed curiosity regarding the rationale for specific measurements: “*That was probably another one that I thought ‘oh, like I wonder why they’re taking that?’ Like what the rationale was for having your arms done*” (P09). Commenting on the adiposity measures via ultrasound scan, one participant commented “*I didn’t really understand it*” (P11). Another participant described a disconnect between method and purpose of the subjective body shape assessment tool, stating it felt “*a bit rudimentary… just for what was happening*” (P07).

#### 3.6.2. Expectations of Follow-Up

Several participants expressed expectations of ongoing measurements or follow-up, indicating a lack of clarity on how the intervention was structured, or intended to function. For some, the absence of continuity created confusion about its purpose: “*I assumed you would do a sort of follow up like a comparison because* […] *I thought it was to sort of track the journey of somebody’s sort of pregnancy rather than just this is what your initial measurements are*.” (P03)

### 3.7. Opportunity Cost

#### Time Pressure vs. Entitlement to Paid Antenatal Leave

Opportunity cost was not a prominent theme for most participants. One participant noted that *“the fact that antenatal care is kind of covered legally, in terms of time off from work, adding 10, 15 min is not like a big deal”* (P03). However, for another, time pressures remained a consideration: *“as soon as you see that a time frame, you’re like, oh God, you know when you’ve got to go back to work, that that would be the only thing that would have put me off”* (P04).

### 3.8. Perceived Effectiveness

Two subthemes related to perceived effectiveness: the personal relevance of the measurements and the role of trust in healthcare professionals.

#### 3.8.1. Personal Relevance

Participants emphasised the importance of the intervention having a clear and meaningful purpose. Perceived effectiveness was not only linked to clinical or measurable outcomes but also to whether the intervention felt worthwhile and relevant to their personal care experience. As one participant reflected, “*What does it do for that woman in* […] *her care? Because that’s the most important thing* […] *that woman has to feel like it was done for a reason.* […] *For some women, they may not have been offered help or know where to start with things and this may be a great opportunity to do that.*” (P07), illustrating future success of changes to routine maternity care would need to clearly articulate benefits beyond clinical utility.

#### 3.8.2. Trust in Healthcare Professionals

At the same time, some participants appeared to equate perceived effectiveness with trust in healthcare professionals. As one primipara explained, “*I think when you’re pregnant, you’re open to absolutely everything and anything that the medical departments are going to offer. I personally was.* […] *they know what they’re doing and they know what they’re looking for*” (P03).

### 3.9. Self-Efficacy

Two subthemes related to self-efficacy were identified: confidence in performing required actions and difficulty in subjective self-assessment. Participants did not express difficulty in completing the anthropometric measurements when clear instructions were provided: “*I just sort of went and just done as I was told*” (P05). However, difficulties related to personal interpretation or self-assessment of body shape were constructed as a sub-theme. While this reflected affective responses for some, most participants indicated a difficulty in their ability to select accurately: “*I’m a bit more top heavy, but I didn’t feel like that was, like, on the pictures, if that makes sense. So it is a bit more like ‘oh, I have to pick one, but I don’t know which one I am’ and trying to push yourself into that picture bracket. That was, yeah, a bit more difficult*” (P04). Some also reflected that their difficulties related to body changes during pregnancy: “*It’s hard to sort of put yourself in a category because* […] *You’re not how you were, but you’re not really different*” (P06).

### 3.10. Design and Delivery Context

Some practical and environmental factors that influenced the experience of the intervention were not directly captured by the TFA but provide important contextual insight into acceptability in practice. These included: physical characteristics of the setting; the non-clinical ‘feel’ of the measurement environment; and staff qualifications and demeanour.

#### 3.10.1. Characteristics of the Setting

Participants generally found the room size small but adequate for measurement purposes. However, some noted limitations for the presence of a partner/chaperone: “*But I don’t think there would have been room for the three of us to be in the room*” (P04). Room temperature was typically viewed as acceptable, particularly in relation to the need to undress: “*It wasn’t too hot, wasn’t too cold. It was, it was perfect for me to be in the appropriate sort of state of dress or undress for*” (P010). However, relocating from the scanning room was considered inconvenient by some: “*I think the only bit for me was a moving between the different places,* […] *But that was more of a logistics thing*” (P11).

#### 3.10.2. Clinical vs. Non-Clinical ‘Feel’ of the Measurement Environment

Participants also identified a contrast in the atmosphere of the measurement room compared to other clinical settings: *“So, it felt clinical and, like getting the scan taken, and then it felt clinical getting my bloods taken. And then in that room it just felt a bit separate” (P06); “It wasn’t as, not inviting, but it didn’t feel as, like, warm an environment” (P11);* and *“But if it was to be integrated into the daily thing, it would be in the clinic room just to make it feel a bit more… make part of your appointment”* (P04).

#### 3.10.3. Staff Qualifications and Demeanour

When asked about whether the anthropometrist met any particular criteria, participants considered that professional healthcare registration was not necessary, but that task-related training would be sufficient, along with displaying the necessary personal qualities for interaction with the public in a healthcare role: “*I just think as long as they’ve had appropriate training in doing that job, and like awareness of the sensitivities involved. I would think that would be OK, but I would say, you wouldn’t, you certainly wouldn’t have to be like a registered healthcare professional to do it*” (P13).

Overall, when asked how other women might respond if these measurements became a routine component of maternity care, participants expressed confidence that most would find them acceptable, particularly if delivered sensitively and integrated smoothly into existing appointments. One participant encapsulated the broader tone of responses: “*I personally feel like you should be fine, and accepted and assume most women would be. I can’t really imagine anyone not being comfortable*” (P03).

## 4. Discussion

This study provides insights into the perceived acceptability of new adiposity measurements undertaken during early pregnancy routine antenatal care. An in-depth exploration of factors known to influence the acceptability of healthcare interventions was possible through the use of the TFA [29].

Results suggest that extending the adiposity measurements within routine maternity care was considered acceptable to women. However, findings illustrate how pregnant women’s affective and cognitive responses to additional adiposity measures are embedded in personal, psychological, and contextual factors. Participants expressed openness to using different or more detailed methods for assessing body composition during pregnancy, alongside clear views on what made these acceptable or burdensome in practice. These findings aligned with Bombard et al.’s [31] conclusion that patient engagement can improve healthcare interventions by informing discrete products and structural aspects of care. In the present study, participants’ suggestions, such as the need for clear pre-appointment materials and communication strategies that emphasise the purpose and benefits of measurements, reflect engagement with discrete products. Meanwhile their emphasis on consent-seeking behaviours, choice regarding partner presence and anthropometrist gender, and the need for private measurement environments illustrates recommendations related to care processes and structural outcomes.

In the present study, some women expressed concerns about judgement, both perceived and internalised, about body image, measurement procedures, and the presence of others. For a few, the act of being measured could prompt some discomfort or self-consciousness, especially when body size deviated from personal or societal expectations. Others described moments of unease at having their bodies exposed or touched. These perceptions are consistent with previous work showing that pregnancy can heighten body image dissatisfaction [16,17,20,21]. Our findings add to this literature by suggesting that the measurement process itself has the potential, in certain circumstances, to act as a trigger for discomfort, particularly if privacy and consent are not prioritised. Measurement interventions can inadvertently reinforce normative ideals and expose women to subtle forms of surveillance or scrutiny. Indeed, in a qualitative study of privacy in perinatal services [32], the authors found that body shape changes during pregnancy, particularly when visible to others, led to a sense of being perceived as pitiable and a desire to hide the body, which may have undermined self-esteem. Yuill et al. [33], in their systematic review of women’s experiences of decision-making and informed choice during pregnancy, comment that “*good motherhood continues to be heavily associated with concepts of the appropriate maternal body*”. Importantly, these concerns are not universal, but it is essential that measurement practices are approached with sensitivity, ensuring that autonomy, consent, emotional safety and privacy are prioritised to avoid reinforcing narrow ideals of the maternal body and to support genuinely person-centred care.

A notable pattern within the data suggested that participants’ experiences of pregnancy and maternity care played a key role in shaping how they perceived the physical contact involved in the measurements. Since the qualitative interviews were undertaken up to 5 months after the measurement experience, participants had navigated many more bodily exposures and procedures associated with pregnancy, routine antenatal care and, for multiparous women, labour. The language used frequently normalised the experience of being touched, referring to being ‘poked and prodded’ as a standard and largely unremarkable part of maternity care. This highlights how embodied knowledge [34], developed through repeated exposure to clinical procedures, may act as a buffer to perceived burden, suggesting that parity could be an important contextual factor influencing the acceptability of new procedures involving physical contact during pregnancy. This contrasts with earlier research, where midwives reported anxiety about causing embarrassment when palpating or measuring women with obesity [23,24]. Our findings highlight a potential mismatch; while professionals may anticipate high burden, women often integrated such procedures into the broader maternity care experience.

Women’s greatest difficulties arose in relation to actively participating in the body assessment process through the use of the subjective body shape tools, consistent with findings from previous studies in the field of body image perception which demonstrate body image distortion in healthy adults [35,36]. Given that women’s bodies undergo rapid changes in size and shape during pregnancy, these tools would need to have high accuracy for risk prediction, currently being examined via the SHAPES study [13], before such tools could be confidently implemented in clinical practice.

The primary purpose of the SHAPES study was to explore the potential of early pregnancy adiposity measures to identify women at higher risk of adverse pregnancy outcomes [13]. The expectation of follow up expressed by participants in the current study suggests that if alternative measurements are found to be useful, any implementation strategy must include clear communication about the purpose of the measurements. That said, since participants desired body composition assessments to be linked to meaningful support or interventions, enabling active engagement in managing risk rather than feeling passively scrutinised for service planning purposes, it may be helpful to further develop services to connect individuals with targeted interventions or clinical support explicitly to enhance acceptability and avoid distress. In a study exploring the acceptability of a cardiometabolic risk prediction tool in early pregnancy [37], the authors reported that women and healthcare professionals recommended the need for each level of risk be accompanied by a clear management plan. These findings align with broader ethical arguments that risk prediction is only justifiable when results are ‘actionable’ by individuals [38], either to make informed decisions, adopt health-positive behaviours, or access appropriate support. However, assumptions that individuals can easily act on risk information are challenged by evidence of barriers such as low health literacy [39], emotional distress, and social inequalities [40]. This underscores the importance of clear, contextualised communication and the need for healthcare professionals to be trained not only in undertaking measurements, but also in supporting women to understand and respond to risk information in ways that feel relevant and manageable.

The findings of this study provide some insight on the acceptability of proposed adiposity assessments and guidance for future implementation of anthropometry-based risk stratification in pregnancy. Ethical implementation should prioritise clear communication, active consent to various steps of measurement, respect for bodily autonomy, and be supported by appropriate staff training.

It is important to note that the SHAPES study [13] included a wide range of anthropometric and body composition measures, and it remains unclear which specific measurements will ultimately prove most useful for risk prediction in early pregnancy. Consequently, the practical implications and acceptability concerns identified in this study may vary depending on which measures are adopted in routine care; if only a limited number of anthropometric measures or ultrasound-based assessments are implemented, some logistical challenges, such as transitioning between clinical areas or longer appointment times caused by multiple procedures, may be mitigated or eliminated. Additionally, while this study was embedded within routine antenatal clinics to approximate real-world care pathways, the research context itself may have influenced participant experiences. Therefore, ongoing evaluation in true clinical practice settings will be essential to fully understand acceptability and optimise implementation strategies.

### Study Strengths and Limitations

This study’s strengths include the use of maximum-variation sampling which facilitated representation of diverse perspectives across BMI, age, and parity. However, while the sample broadly reflected the larger cohort’s demographic composition, it remained predominantly White, limiting ethnic diversity. This may reduce the transferability of findings, as culture and religious beliefs may influence attitudes towards bodily exposure, measurement and instrumental touch [41]. The sample size (n = 14) aligns with Hennink & Kaiser’s [42] guidance that, in studies with focussed aims and relatively homogenous samples, thematic saturation is often reached within a small number of interviews (typically 9–17). Saturation was indicated by diminishing new insights in the later interviews, suggesting sufficient data to capture key themes. A structured, yet flexible, interview guide, informed by expert input and anthropometric standards, supported in-depth exploration of participants’ views. Thematic analysis was enhanced by independent coding and use of the TFA [29], adding rigour and depth. While this framework was useful for structuring individual-level responses and appropriate for the current study aim, it does not explicitly account for wider systemic or organisational influences that may also shape the acceptability of new antenatal measurements. This study focused solely on women’s views; professional perspectives (e.g., midwives and sonographers) are also crucial for assessing feasibility and sustainability but could not be included as SHAPES measurements were undertaken predominantly by one researcher, with limited contributions from five other anthropometrists, none of whom were midwives or sonographers. Reflexivity and transparent reporting further strengthened the trustworthiness of the findings. However, the use of a probing interview approach, while valuable in eliciting rich and nuanced insights, may have amplified concerns that were not strongly felt by most participants. Consequently, the thematic depth achieved should be interpreted within the broader context of generally neutral or modestly positive attitudes. Interviews were conducted approximately 4–5 months after participants’ routine antenatal ultrasound scan appointments introducing potential recall bias and normalisation of bodily exposure due to intervening antenatal experiences. Nonetheless, the temporal distance may have allowed participants to reflect more broadly on the significance and relevance of the experience within the context of their ongoing pregnancy care. For some specific elements, such as being comfortable with male staff taking the measures, post hoc rationalisation may have occurred, especially given the minimally invasive nature of the measurements. Anticipated discomfort may still exist prior to exposure, particularly among first-time mothers. No contemporaneous data sources such as diaries or direct observations were collected; therefore, triangulation was not possible. This may have mitigated recall bias and strengthened interpretation of participants’ accounts. Finally, self-selection bias may have limited the perspectives represented. Women who agreed to participate in a study involving additional adiposity measures, and had sufficient English language to participate in interviews, may differ from those who declined, potentially under-representing those with lower health-literacy or body-positivity, language-related communication barriers, or different cultural or religious attitudes towards body measurement. Should SHAPES findings support the incorporation of additional body measures into routine care, further research will be needed to capture the perspectives of women beyond those represented in the SHAPES cohort, including whether views differ across groups such as those defined by BMI category.

## 5. Conclusions and Recommendations

By directly addressing women’s perceptions, experiences, and retrospective acceptability of adiposity measures, this study fills a gap in the literature that has largely centred on professional perspectives. The findings of this study suggest that expanding maternal adiposity measurements to include a broader range of anthropometric and body composition measures is acceptable to pregnant women when delivered with sensitivity, clarity and respect for autonomy. Overall, no major concerns were raised, and participants generally viewed the additional measurements as acceptable, particularly when integrated into routine antenatal care. Acceptability was enhanced by emotional comfort, perceived relevance, and the quality of communication. For ethical implementation, women should be offered practical pre-appointment guidance on clothing, and healthcare staff should demonstrate good communication, attention to privacy, and consent-seeking behaviour throughout.

## Figures and Tables

**Table 1:** Theoretical Framework of Acceptability [29] Constructs and Definitions [adapted].

Construct	Definition
Affective attitude	How an individual feels about the intervention (i.e., measurements)
Burden	The perceived amount of effort required to engage with the measurements
Ethicality	The extent to which the measurements align with an individual’s value system
Intervention coherence	The extent to which the individual understands the measurements and how they work
Opportunity costs	The extent to which benefits, values, or profits must be given up to engage in the measurements
Perceived effectiveness	The extent to which the measurements are perceived as likely to achieve their purpose
Self-efficacy	The individuals’ confidence that they can participate in the measurements

**Table 2:** Participant demographics.

ID	Age (y)	Ethnicity	Previous Pregnancies	Parity ^1^	BMI ^2^ (kg/m^2^)	IMD Decile ^3^
P01	30	White	2	0	33.0	4
P02	34	Black	1	1	33.8	4
P03	31	Mixed	0	0	35.6	9
P04	34	White	2	1	27.3	10
P05	31	White	2	0	25.1	7
P06	26	White	2	2	22.0	4
P07	35	White	2	1	23.7	9
P08	34	White	0	0	24.9	8
P09	33	White	1	1	34.8	1
P10	34	White	0	0	20.8	7
P11	30	White	1	1	30.0	2
P12	33	Asian	0	0	24.7	2
P13	34	White	0	0	17.4	5
P14	35	White	3	2	20.8	3

^1^ parity = the number of previous pregnancies >24 gestation; ^2^ BMI: Body mass index; ^3^ IMD: Index of multiple deprivation (1 = most deprived).

**Table 3:** Themes and subthemes.

Theme	Subthemes
Affective attitude	Positive orientation towards enhanced care Sensory and emotional responses to measurement procedures Internal discomfort: body image and vulnerability Contextual discomfort: haptics and proxemics
Burden	Physical effort Time commitment and waiting Privacy
Ethicality	BMI as ethically problematic Choice, autonomy and consent
Intervention coherence	Measurement purpose Expectations of follow-up
Opportunity cost	Time pressure vs. entitlement to paid antenatal leave
Perceived effectiveness	Personal relevance Trust in healthcare professionals
Self-efficacy	Confidence in performing required actions Difficulty in subjective self-assessment
Design and delivery contexts *	Characteristics of the setting Clinical vs. non-clinical ‘feel’ of the measurement environment Staff qualifications and demeanour

* inductive theme.

## Data Availability

The raw data supporting the conclusions of this article will be made available by the authors on request.

## Supplementary Materials

The following supporting information can be downloaded at https://www.mdpi.com/article/10.3390/healthcare13202558/s1: Document S1: Topic Guide. Table S1: SRQR guidelines [30]. Table S2: Minimal Dataset.

## Abbreviations

The following abbreviations are used in this manuscript:

BMI	Body Mass Index
IMD	Indices of Multiple Deprivation
NHS	National Health Service
NICE	National Institute for Health and Care Excellence
SHAPES	Study of How Adiposity in Pregnancy has an Effect on outcomeS
SRQR	Standards for Reporting Qualitative Research
TFA	Theoretical Framework of Acceptability

## References

[1] National Institute for Health and Care Excellence (2025). Nice Guideline NG246: Overweight and Obesity Management. https://www.nice.org.uk/guidance/ng246/resources/overweight-and-obesity-management-pdf-66143959958725.

[2] Simpson F., Doig G.S., Early PN Trial Investigators Group (2015). Physical Assessment and Anthropometric Measures for use in Clinical Research Conducted in Critically Ill Patient Populations: An Analytic Observational Study. J. Parenter. Enter. Nutr..

[3] Pan S., Zhu L., Chen M., Xia P., Zhou Q. (2016). Weight-based Dosing in Medication Use: What Should We Know?. Patient Prefer. Adherence.

[4] National Institute for Health and Care Excellence (2021). Nice Guideline NG201: Antenatal Care. https://www.nice.org.uk/guidance/ng201/resources/antenatal-care-pdf-66143709695941.

[5] National Institute for Health and Care Excellence (2015). Nice Guideline NG3: Diabetes in Pregnancy: Management from Preconception to the Postnatal Period. https://www.nice.org.uk/guidance/ng3/resources/diabetes-in-pregnancy-management-from-preconception-to-the-postnatal-period-pdf-51038446021.

[6] Denison F., Aedla N., Keag O., Hor K., Reynolds R., Milne A., Diamond A. (2019). Care of Women with Obesity in Pregnancy. Bjog.

[7] National Institute for Health and Care Excellence (2019). Nice Guideline NG121: Intrapartum Care for Women with Existing Medical Conditions or Obstetric Complications and Their Babies. https://www.nice.org.uk/guidance/ng121.

[8] Public Health England (2019). Health of Women before and During Pregnancy: Health Behaviours, Risk Factors and Inequalities. An Updated Analysis of the Maternity Services Dataset Antenatal Booking Data.

[9] Okorodudu D.O., Jumean M., Montori V.M., Romero-Corral A., Somers V.K., Erwin P.J., Lopez-Jimenez F. (2010). Diagnostic Performance of Body Mass Index to Identify Obesity as Defined by Body Adiposity: A Systematic Review and Meta-Analysis. Int. J. Obes..

[10] Janssen I., Katzmarzyk P.T., Ross R. (2004). Waist Circumference and Not Body Mass Index Explains Obesity-Related Health Risk. Am. J. Clin. Nutr..

[11] Heslehurst N., Ngongalah L., Bigirumurame T., Nguyen G., Odeniyi A., Flynn A., Smith V., Crowe L., Skidmore B., Gaudet L. (2022). Association Between Maternal Adiposity Measures and Adverse Maternal Outcomes of Pregnancy: Systematic Review and Meta-analysis. Obes. Rev..

[12] Saw L., Aung W., Sweet L. (2021). What are the Experiences of Women with Obesity Receiveing Antenatal Maternity Care? A Scping Review of Qualitative Evidence. Women Birth.

[13] Heslehurst N., Vinogradov R., Nguyen G.T., Bigirumurame T., Teare D., Hayes L., Lennie S.C., Murtha V., Tothill R., Smith J. (2023). Study of How Adiposity in Pregnancy Has an Effect on Outcomes (SHAPES): Protocol for a Prospective Cohort Study. BMJ Open.

[14] Sangkum L., Klair I., Limsuwat C., Bent S., Myers L., Thammasitboon S. (2017). Incorporating Body-Type (Apple Vs. Pear) in Stop-Bang Questionnaire Improves Its Validity to Detect Osa. J. Clin. Anesth..

[15] Thoma M.E., Hediger M.L., Sundaram R., Stanford J.B., Matthew Peterson C., Croughan M.S., Chen Z., Louis G.M.B. (2012). Comparing Apples and Pears: Women’s Perceptions of Their Body Size and Shape. J. Women’s Health.

[16] Hill I.F., Angrish K., Nutter S., Ramos-Salas X., Minhas H., Nagpal T.S. (2023). Exploring Body Dissatisfaction in Pregnancy and the Association with Gestational Weight Gain, Obesity, and Weight Stigma. Midwifery.

[17] Rodgers R.F., Campagna J., Hayes G., Sharma A., Runquist E., Fiuza A., Coburn-Sanderson A., Zimmerman E., Piran N. (2024). Sociocultural Pressures and Body Related Experiences During Pregnancy and the Postpartum Period: A Qualitative Study. Body Image.

[18] Loth K.A., Bauer K.W., Wall M., Berge J., Neumark-Sztainer D. (2011). Body Satisfaction During Pregnancy. Body Image.

[19] Padmanabhan U., Summerbell C.D., Heslehurst N. (2015). A Qualitative Study Exploring Pregnant Women’s Weight-Related Attitudes and Beliefs in Uk: The Bloom Study. BMC Pregnancy Childbirth.

[20] Zaltzman A., Falcon B., Harrison M.E. (2015). Body Image in Adolescent Pregnancy. J. Pediatr. Adolesc. Gynecol..

[21] Downs D.S., DiNallo J.M., Kirner T.L. (2008). Determinants of Pregnancy and Postpartum Depression: Prospective Influences of Depressive Symptoms, Body Image Satisfaction, and Exercise Behavior. Ann. Behav. Med..

[22] Rallis S., Skouteris H., Wertheim E.H., Paxton S.J. (2007). Predictors of Body Image During the First Year Postpartum: A Prospective Study. Women Health.

[23] Christenson A., Johansson E., Reynisdottir S., Torgerson J., Hemmingsson E. (2018). Shame and Avoidance as Barriers in Midwives’ Communication About Body Weight with Pregnant Women: A Qualitative Interview Study. Midwifery.

[24] Heslehurst N., Russell S., McCormack S., Sedgewick G., Bell R., Rankin J. (2013). Midwives Perspectives of Their Training and Education Requirements in Maternal Obesity: A Qualitative Study. Midwifery.

[25] Atkinson S., McNamara P.M. (2017). Unconscious collusion: An interpretative phenomological analysis of the maternity care experiences of women with obesity (BMI ≥ 30 kg/m^2^). Midwifery.

[26] Denicolo P., Bradley-Cole K., Long T. (2016). Constructivist Approaches and Research Methods: A Practical Guide to Exploring Personal Meanings.

[27] Braun V., Clarke V. (2013). Successful Qualitative Research: A Practical Guide for Beginners.

[28] Esparza-Ros F., Vaquero-Cristóbal R., Marfell-Jones M. (2019). International Standards for Anthropometric Assessment.

[29] Sekhon M., Cartwright M., Francis J.J. (2017). Acceptability of Healthcare Interventions: An Overview of Reviews and Development of a Theoretical Framework. BMC Health Serv. Res..

[30] O’Brien B.C., Harris I.B., Beckman T.J., Reed D.A., Cook D.A. (2014). Standards for Reporting Qualitative Research: A Synthesis of Recommendations. Acad. Med..

[31] Bombard Y., Baker G.R., Orlando E., Fancott C., Bhatia P., Casalino S., Onate K., Denis J.-L., Pomey M.-P. (2018). Engaging Patients to Improve Quality of Care: A Systematic Review. Implement. Sci..

[32] Aksoy S., Komurcu N. (2018). Privacy in Perinatal Services: A Qualitative Study. Nurs Health Sci.

[33] Yuill C., McCourt C., Cheyne H., Leister N. (2020). Women’s Experiences of Decision-Making and Informed Choice About Pregnancy and Birth Care: A Systematic Review and Meta-Synthesis of Qualitative Research. BMC Pregnancy Childbirth.

[34] Abel E.K., Browner C.H., Lock A.M., Kaufert P. (1998). Selective Compliance with Biomedical Authority and the Uses of Experiential Knowledge. Pragmatic Women and Body Politics.

[35] Fuentes C.T., Longo M.R., Haggard P. (2013). Body Image Distortions in Healthy Adults. Acta Psychol..

[36] Gruszka W., Owczarek A.J., Glinianowicz M., Bąk-Sosnowska M., Chudek J., Olszanecka-Glinianowicz M. (2022). Perception of Body Size and Body Dissatisfaction in Adults. Sci. Rep..

[37] Lang S., McIntosh J.G., Enticott J., Goldstein R., Baker S., McGowan M., Cooray S., Du L., Reddy A., Harrison C.L. (2025). Exploring the Acceptability of a Risk Prediction Tool for Cardiometabolic Risk (Gestational Diabetes and Hypertensive Disorders of Pregnancy) for Use in Early Pregnancy: A Qualitative Study. Midwifery.

[38] Jansen S.N., Kamphorst B.A., Mulder B.C., van Kamp I., Boekhold S., van den Hazel P., Verweij M.F. (2024). Ethics of Early Detection of Disease Risk Factors: A Scoping Review. BMC Med. Ethics.

[39] Damman O.C., Bogaerts N.M., van Dongen D., Timmermans D.R. (2016). Barriers in Using Cardiometabolic Risk Information among Consumers with Low Health Literacy. Br. J. Health Psychol..

[40] Janßen C., Sauter S., Kowalski C. (2012). The Influence of Social Determinants on the Use of Prevention and Health Promotion Services: Results of a Systematic Literature Review. GMS Psycho-Soc.-Med..

[41] Buono R.A., Nygren M., Bianchi-Berthouze N. (2025). Touch, Communication and Affect: A Systematic Review on the Use of Touch in Healthcare Professions. Syst. Rev..

[42] Hennink M., Kaiser B.N. (2022). Sample Sizes for Saturation in Qualitative Research: A Systematic Review of Empirical Tests. Soc. Sci. Med..

