# Supplementary material for: Exploring Pregnant Women’s Perceptions and Experiences of Adiposity Measurements in Routine Antenatal Care: A Qualitative Study
Source: Healthcare (Basel). 2025 Oct 10;13(20):2558. doi: 10.3390/healthcare13202558 (PMC12562455; doi:10.3390/healthcare13202558)
Supplement: Supplementary file 1 [file healthcare-13-02558-s001.zip › Document S1 Topic Guide.pdf]

Document S1:Topic Guide

Establishing Rapport
You took part in the SHAPES study just in the last few months, so when is your baby due?
Is this your first child?

I'm going to ask questions in the following areas:
Previous body measurement experiences
Information received as part of the study, and your expectations of what might happen during the appointment.
Measurement environment
Your experience of being measured
And your thoughts on measurements being part of routine care.

I'll try to ask open questions, so feel free to just share whatever comes to mind. But I might ask some follow up questions, or prompt you to share your thoughts on specific aspects as we go through. In some areas, it might feel link the questions are similar or a bit repetitive, but I'll just be trying to be sure that I've understood your thoughts, and exploring them from different angles for the purposes of the research.

Are you ready to begin?

Was there any particular reason that you wanted to take part in the SHAPES study?

1. Previous body measurement experiences
Can you tell me about any past experience of body measurements that you had before taking part in the SHAPES study?
If yes, where, when and by whom? For what purpose?
Prompts:
- Do you take any body measurements regularly yourself? E.g. weight. If so, how frequently?
- Had you ever had skinfold measurements taken before participating in the study. If so, where, when and by whom? For what purpose?
- Circumferences

2. Information and Expectations
Before you came to the appointment, what were your expectations about the measurements?
Prompts:
- What did you think about the information that you received?
- What was particularly clear?
- Could anything have been clearer beforehand?
- Did you ask questions?

When you came to the appointment, was any further information given to you about what the measurements would involve?
Prompts:
- If yes, what were you told?
- If no, is there anything else that would have been useful to be told at the start of the appointment?

3. Measurement Environment
How did you feel about the measurement environment?
Prompts:
- Was the venue private enough?

- Was there enough space for taking the measurements?
- Was the measurement room a comfortable temperature?
- Did you have anyone else in the room with you? Were you offered the option of having someone with you? Did you feel it was important to have someone else there with you? Or do you think that
- having someone with you would be important?

4. Experience of the measurements

Measurement acceptability

How did you feel about having the measurements taken?

Prompts: Do you remember having any of the following measurements taken?

- Height
- Weight
- Waist circumference
- Hip circumference
- Neck size
- Arm circumference
- Skinfold measures
    - Front of the arm (biceps)
    - Back of the arm (triceps)
    - Back of shoulder (subscapular)
    - Side of waist (Iliac crest)
    - Front of hip (Iliospinale)
- Subjective Measures
- US scan measurements

Were you comfortable with some measures more than others?

Which measurements did you like, or find more comfortable?

Which measurements didn't you like, or found less comfortable?

Did having the measurement taken raise any concerns for you? Did you discuss these concerns with the SHAPES team? Why/why not? Was there someone else you discussed them with?

Haptics

Having the measurements required someone to touch you and draw marks on your skin. How did you feel about this aspect?

Prompts:

- Were you asked to remove or adjust any items of clothing to enable the measurements to be taken?
    - How did you feel about this?
- Did you have any concerns about having the marks made on your skin?
    - If yes, what were your concerns?
    - How were these dealt with in the appointment?

Measurement Etiquette

What was your experience of the person undertaking the measurements?

Prompts:

- Did the measurer maintain professionalism throughout? If not, what happened? If yes, how was that demonstrated to you?
- Was the measurer sensitive to any concerns you had about being measured? Tell me more about this.
- Did they explain what they were going to measure before each measurement?

5. Future Implementation

If these measurements were to become a routine part of maternity care appointments, how do you think women will feel about this?
Prompts:

- Is there any information that you feel women might need to know?
- Are there any measures they might be more ok with than others?
- Do you think there will be any problems with doing these measurements in routine care?
    - If no, why not?
    - If yes, what might these be?
        - How do you think we might be able to overcome these problems?
- Would you recommend that that person taking the measurement met any particular criteria?
- Would you make any recommendations for the environment to be measured?

6. Conclusion
Leading up to this interview, was there anything that you expected to talk about today that we didn't discuss?
Is there anything else that you would like to comment on that I haven't already asked you about?

Thank you very much for your time and the information you shared today.
