# Supplementary material for: Exploring Pregnant Women’s Perceptions and Experiences of Adiposity Measurements in Routine Antenatal Care: A Qualitative Study
Source: Healthcare (Basel). 2025 Oct 10;13(20):2558. doi: 10.3390/healthcare13202558 (PMC12562455; doi:10.3390/healthcare13202558)
Supplement: Supplementary file 1 [file healthcare-13-02558-s001.zip › healthcare-3769130-table S2.pdf]

Table S2: Minimal Dataset

Inductive Code	Unique Participants	Occurrences
Acceptability of time spent on measurements	1	2
Acceptable	9	23
Acceptance in healthcare	2	2
Acceptance of physical invasions in pregnancy care	9	16
Actionable research or changing practice	1	4
Activity changes in pregnancy	2	2
Advanced notice	1	1
Altruism	4	8
Amusing	1	3
Anthropometrist behaviour	13	41
As expected	9	20
Avoid reminders	1	1
Avoid weight	4	5
Awareness of personal BMI status	4	7
Baby size not linked to mothers size	1	2
Basic	1	1
Being measured before - clothes	3	3
Being measured before - healthcare	8	16
Being measured before - research	1	1
Being measured before - School	1	2
Being measured before - slimming groups	1	2
Being measured before Gym	9	20
Being measured before Self-monitoring	11	18
Belief in improving measurement practices	1	2
Belongings storage	3	4
Benefit	8	17
Bia	1	1
BMI isn't good	7	15
BMI misclassifying risk	3	6
BMI relationship to pregnancy	1	1
Body dissatisfaction	1	1
Body position	2	2
Body shape generalisations	1	1
Body shape perception	11	39
Can't remember	7	15
Chaperone automatic attendance	8	10
Chaperone may be needed	8	13
Chaperone not needed	7	9
Chaperone not offered	3	4
Chaperone not present	3	4
Chaperone offered	3	3
Choice and autonomy in care	11	23
Clear explanations given	4	10
Clothing	5	7
Clothing adjustment	12	46
Comfort	4	5
Comfort during measurement process	2	4

Comfortable	6	12
Communication style	7	19
Concerns over sensitivity to certain measures	2	2
Confidentiality	1	1
Consent	10	16
Continuity	1	1
Continuity of care	1	1
Critical view of regular measures	1	1
Different from scan	1	1
Difficult controlling body size	1	1
Dignity	1	1
Dimensions vs fat or weight	4	6
Duration - short	9	15
Effects of body weight	1	1
Efficient	6	8
Embarrassment	1	1
Employment law	3	3
Environment - clinical preferred	4	6
Environment - private	14	29
Environment - room size	12	31
Environment - temperature	11	14
Environment big enough	10	16
Environment non-clinical	5	13
Environment temperature preference	1	1
Environment too hot	2	4
Equipment accessibility	2	3
Equipment familiarisation	2	3
Ethnicity	2	4
Expectations discrepancy	2	3
Expectations of results - support women who are obese	4	9
Expectations vs. Experience	3	5
Experience of anthropometrist	1	1
Exposed	1	1
Future	2	4
Gender preference - female	4	7
Gender sensibility - personally don't mind	9	15
Gender sensitivity in care provision - others may mind	9	15
Girths okay	1	1
Giving back	1	2
Haptics	12	14
HCP expected	2	4
HCP not required	12	17
HCP required	1	1
Healthcare Assistant acceptable	1	1
Husband or Partner	12	34
Important for females	1	1
Improve care	2	2
Improve knowledge and understanding	1	2
Increase environment size	1	1
Individuality of experience	10	25
Info available	2	3

Info clear	4	11
Information clarity	7	14
Information overload	2	5
Informed consent	3	5
Instructions given	1	1
Insufficient measures	1	1
Interested	1	2
Intimate	3	3
Judgement	5	7
Knowledgeable	2	2
Lacked awareness of measures	1	1
Less information required in routine care	1	1
Location	5	7
Measurement difficulties	1	3
Minimal discomfort	1	1
Motivation - contributing to research	9	11
Motivation - curiosity	1	1
Motivation - improve maternal care	1	1
Motivation - personal body size	1	1
Motivation - science	1	1
Multiparous	1	1
Navigational challenges	3	4
New experience	11	21
No concerns	11	26
No expectations	1	2
No pain or discomfort	9	24
No window	1	1
Non-invasive	4	7
Not problematic	1	1
Not self-conscious	1	4
Not surprised	1	1
Nutrition important	1	2
Obesity disease risks	1	1
Obesity problem	1	1
Objective measure factual	1	1
Odd	1	2
Openness to care in pregnancy	2	5
Overlooking serious health conditions	1	2
Pen marks	12	23
Personal comfort with physical contact	2	2
Personal health	3	4
Personal independence	1	2
Physical discomfort	6	13
Poor maternity care	1	5
Professional	1	1
Positive experience	4	9
Pregnancy adaptation normalisation	2	5
Pregnancy outcomes	1	1
Pregnancy weight gain	7	16
Preparation needed	9	17
Previous measures - tape measure	10	12

Previous measures - weight and height	9	11
Previous measures skinfolds	4	7
Probably	1	1
Proxemics	1	1
Psychological discomfort	8	21
Purpose - tracking	6	10
Questions	10	19
Rationale	8	24
Read info	2	4
Received enough info	1	1
Reiterated info	11	32
Religion	2	2
Repeat measures	5	15
Resource limitations	7	7
Respectful	1	2
Room size small	7	10
Safety	4	6
Scheduling	2	7
Self-perception	2	2
Self-conscious	5	6
Sensation, feelings	9	27
Separate room	2	2
Skepticism of generalisations	1	1
Skinfolds unacceptable	1	1
Skinfolds unexpected	2	2
Stage of pregnancy	3	5
Stoicism	1	2
Suggestion for improving environment - temperature	1	1
Support for routine implementation	4	4
Support women to change	2	9
Surprise	1	1
Task related training	8	10
Time	12	31
Trust in medical professionals	4	7
Uncertain expectations	1	1
Unexpected	3	3
Unquestioning acceptance	3	7
Us scans	4	9
Validity	3	7
Varied needs and wants	1	1
Volume of measures	1	4
Vulnerable	1	3
Waiting time	5	12
Weight not reliable	2	2
Weight stigma	5	13
Window	2	4
